# Pharmacokinetic/Pharmacodynamic Based Breakpoints of Polymyxin B for Bloodstream Infections Caused by Multidrug-Resistant Gram-Negative Pathogens

**DOI:** 10.3389/fphar.2021.785893

**Published:** 2022-01-04

**Authors:** Xingchen Bian, Xiaofen Liu, Fupin Hu, Meiqing Feng, Yuancheng Chen, Phillip J. Bergen, Jian Li, Xin Li, Yan Guo, Jing Zhang

**Affiliations:** ^1^ Institute of Antibiotics, Huashan Hospital, Fudan University, Shanghai, China; ^2^ Key Laboratory of Clinical Pharmacology of Antibiotics, Shanghai, China; ^3^ National Health Commission & National Clinical Research Center for Aging and Medicine, Huashan Hospital, Fudan University, Shanghai, China; ^4^ Department of Biological Medicines & Shanghai Engineering Research Center of Immunotherapeutics, School of Pharmacy, Fudan University, Shanghai, China; ^5^ Phase I Unit, Huashan Hospital, Fudan University, Shanghai, China; ^6^ Biomedicine Discovery Institute and Department of Microbiology, Monash University, Melbourne, VIC, Australia

**Keywords:** susceptibility breakpoint, polymyxin B, PK/PD analysis, Monte-Carlo simulation, bloodstream infection

## Abstract

The latest PK/PD findings have demonstrated negligible efficacy of intravenous polymyxins against pulmonary infections. We investigated pharmacokinetic/pharmacodynamic (PK/PD)-based breakpoints of polymyxin B for bloodstream infections and the rationality of the recent withdrawal of polymyxin susceptibility breakpoints by the CLSI. Polymyxin B pharmacokinetic data were obtained from a phase I clinical trial in healthy Chinese subjects and population pharmacokinetic parameters were employed to determine the exposure of polymyxin B at steady state. MICs of 1,431 recent clinical isolates of *Pseudomonas aeruginosa*, *Acinetobacter baumannii* and *Klebsiella pneumoniae* collected from across China were determined. Monte-Carlo simulations were performed for various dosing regimens (0.42–1.5 mg/kg/12 h via 1 or 2-h infusion). The probability of target attainment, PK/PD breakpoints and cumulative fraction of response were determined for each bacterial species. MIC_90_ of polymyxin B was 1 mg/L for *P. aeruginosa* and 0.5 mg/L for *A. baumannii* and *K. pneumoniae*. With the recommended polymyxin B dose of 1.5–2.5 mg/kg/day, the PK/PD susceptible breakpoints for *P. aeruginosa, A. baumannii* and *K. pneumoniae* were 2, 1 and 1 mg/L respectively for bloodstream infection. For Chinese patients, polymyxin B dosing regimens of 0.75–1.5 mg/kg/12 h for *P. aeruginosa* and 1–1.5 mg/kg/12 h for *A. baumannii* and *K. pneumoniae* were appropriate. Breakpoint determination should consider the antimicrobial PK/PD at infection site and delivery route. The recent withdrawal of polymyxin susceptible breakpoint by CLSI primarily based on poor efficacy against lung infections needs to be reconsidered for bloodstream infections.

## Introduction

Carbapenem-resistant Gram-negative bacteria are a serious threat to global health. In China, approximately 20–30% of *Pseudomonas aeruginosa* and *Klebsiella* spp. and >70% of *Acinetobacter* spp. are now carbapenem resistant (http://www.chinets.com/). Elsewhere, the World Health Organization has reported high median resistance rates to carbapenems in several bacterial species globally, exemplified by resistance rates in *Klebsiella* spp*.* and *Acinetobacter* spp*.* isolated from bloodstream infections of ∼20 and ∼60%, respectively (2020; https://www.who.int/glass/resources/publications/early-implementation-report-2020/en/). Although the newly developed β-lactam/β-lactamase inhibitor combinations (such as ceftazidime-avibactam and ceftolozane-tazobactam) have mitigated the situation, none of them is active against metallo-β-lactamase-producing *Enterobacterales*, *P. aeruginosa* or carbapenemase-producing *A. baumannii* (Yahav et al., 2020). Given many of these carbapenem-resistant bacteria remain susceptible to polymyxins (polymyxin B and colistin), this once abandoned class of antibiotics is often the only viable treatment option were revived and being listed on the reserve list of antibiotics by WHO (Jian et al., 2019).

The polymyxins are cyclic lipopeptides naturally produced by the Gram-positive *Paenibacillus polymyxa* (Velkov et al., 2019). While various polymyxins have been described, only polymyxin B and E (the latter known as colistin) are available for clinical use (Ross et al., 1959; Clifford and Stewart, 1961). The antimicrobial activity is initiated by an electrostatic attraction, causing displacement of divalent cations (Ca^2+^ and Mg^2+^) that bridge adjacent LPS molecules and resulting in OM leaflet expansion, disruption of the membrane integrity, and increased membrane permeability (Berglund et al., 2015; Rabanal and Cajal, 2017). Prior to 2020, the Clinical and Laboratory Standards Institute (CLSI) provided susceptible and resistant breakpoints for colistin and polymyxin B (e.g., M100-S29). However, in 2020 the susceptible interpretive category (previously ≤2 mg/L in all cases) was removed (M100-S30). In contrast, the European Committee on Antimicrobial Susceptibility Testing (EUCAST) has maintained the “susceptible” category for colistin (polymyxin B breakpoints are not reported) (Version 10.0, 2020). The decision by the CLSI was based primarily on data suggesting that intravenous polymyxins have limited efficacy for the treatment of lung infections in mice and patients (Rigatto et al., 2013; Cheah et al., 2015; Landersdorfer et al., 2018; Satlin et al., 2020). Indeed, studies in animals clearly show reduced bacterial killing in lung infection models compared to thigh infection models with equivalent parenteral dosage regimens (Cheah et al., 2015; Landersdorfer et al., 2018), and substantially lower concentrations of polymyxin B in epithelial lining fluid (ELF) compared to plasma have been reported following intravenous administration (He et al., 2013). Such data clearly suggests relatively low unbound concentrations are achieved in pulmonary fluids (He et al., 2013). Given that higher polymyxin concentrations can be achieved elsewhere in the body (e.g., blood), the CLSI decision to remove the susceptible category for polymyxins based primarily on lung infection data may not be justified for other infection sites such as the bloodstream.

Various historical factors led to colistin being adopted far more widely than polymyxin B. Consequently, most existing polymyxin studies involve colistin and its inactive prodrug, colistin methanesulfonate (CMS) (Tsuji et al., 2019). Recently, however, the use of polymyxin B for treatment of systemic infections has increased, primarily due to better PK characteristics and relatively lower rates of nephrotoxicity than CMS (Aggarwal and Dewan, 2018; Tsuji et al., 2019). Unfortunately, very few clinical studies have utilized polymyxin B (Kvitko et al., 2011; Rigatto et al., 2013; Terayama et al., 2017). Given the circumstances described here, we investigated susceptibility breakpoints for polymyxin B for treatment of bloodstream infections caused by carbapenem-resistant Gram-negative bacteria and the rationality of the recent withdrawal of the ‘susceptible’ category for polymyxins by the CLSI. Clinical breakpoints were determined by epidemiological cut-offs, PK/PD breakpoints and clinical efficacy (Li et al., 2015). To acquire PK/PD breakpoints, we conducted a PK study in healthy Chinese volunteers (Liu et al., 2021) and collected 1,431 isolates of *P. aeruginosa*, *A. baumannii* and *K. pneumoniae* from patients across China for PK/PD analysis. This study will provide useful information of PK/PD breakpoints for polymyxin B in patients and evaluate polymyxin dosing regimens for bloodstream infections caused by different bacterial species.

## Materials and Methods

### Pharmacokinetics of Polymyxin B

A single-center, randomized, open-label phase I clinical trial of intravenous polymyxin B (0.75 and 1.5 mg/kg) were conducted in healthy Chinese subjects (Liu et al., 2021). The liquid chromatography-tandem mass spectrometry (LC-MS/MS) assay of polymyxin B was employed to determine the concentrations as previously reported (Liu et al., 2020). Non-compartment analysis was employed to calculate the AUC_0-inf_ of polymyxin B in WinNonlin (v8.0, Pharsight, United States), which was linear with dose according to a power model with a 95% confidence interval (0.94, 1.2). Population pharmacokinetic (PPK) analysis was conducted using NONMEM 7.4 (Icon Development Solutions, Ellicott City, MD) with G77 FORTRAN complier and FOCEI algorithm. The base model of polymyxin B was fit into a three-compartment model. The interindividual and residual variabilities were best described by an exponential model and a proportional model, respectively. Age and gender were included in the final PK model (Liu et al., 2021).

### Microbiological Information

A total of 1,431 clinical isolates (517 strains of *P. aeruginosa*, 262 of *A. baumannii* and 652 of *K. pneumoniae*) were collected across 2017–2019 from more than 30 teaching hospitals in 23 provinces across China. Polymyxin B and meropenem minimum inhibitory concentrations (MICs) were determined by broth microdilution and interpreted according to the CLSI breakpoints (M100-S30, 2020). *Escherichia coli* ATCC 25922, *E. coli* ATCC 35218 and *K. pneumoniae* ATCC 700603 acted as quality control strains. Statistical analysis was performed using WHONET (version 5.6). The study protocol was approved by the Institutional Review Board of Huashan Hospital, Fudan University (no. 2018-408, no. 2019-460).

### Pharmacokinetic/Pharmacodynamic Targets for Polymyxin B Against the Three Gram-Negative Pathogens

The area under the unbound concentration-time curve over 24 h to the MIC ratio (*f*AUC_0–24h_/MIC) is the most predictive PK/PD index for polymyxins (Tam et al., 2005; Bergen et al., 2008; Bergen et al., 2010; Cheah et al., 2015; Landersdorfer et al., 2018). PK/PD targets of 1-log_10_ CFU and 2-log_10_ CFU reductions in colony forming units (CFU)/thigh were derived from dose-fractionation studies of polymyxin B (*K. pneumoniae*) and colistin (*P. aeruginosa* and *A. baumannii*) in murine thigh infection models (Supplementary Table S1; Cheah et al., 2015; Landersdorfer et al., 2018). Given polymyxin B and colistin have essentially identical *in vitro* potencies and spectra of antibacterial activity (Gales et al., 2011), the colistin targets were adopted for polymyxin B. An unbound fraction (*f*) of polymyxin B in plasma of 42% was applied (Sandri et al., 2013).

### Probability of Target Attainment, Pharmacokinetic/Pharmacodynamic Breakpoints and Cumulative Fraction of Response

Four thousand data sets were generated with the final PPK model based on the estimated PPK parameters for each dosage regimen ranging from 0.42 to 1.5 mg/kg/12 h (administered via a 1- or 2-h infusion). Mean and SD of AUC_0–24,ss_ were calculated using the simulated AUC_0-12,ss_. Monte-Carlo simulations for PTA and CFR were performed using MATLAB (Mathworks, United States, version 7.0.1). The add-in macro permitted the MATLAB program to perform Monte-Carlo simulations for 10,000 simulated data sets (AUC_0–24,ss_ ± SD). The upper limit of the MIC range was taken as the PK/PD breakpoint when PTA was >90%. MIC distributions of polymyxin B for each bacterial species were used to determine the CFR, with a CFR of 90% considered effective.

## Results

### Susceptibility and PK Data

Polymyxin B MIC distributions and MIC_50_/MIC_90_ values for the 1,431 clinical isolates are shown in Table 1. MICs ranged between 0.125 and >32 mg/L for *P. aeruginosa*, 0.25 to >32 mg/L for *A. baumannii*, and 0.25–16 mg/L for *K. pneumoniae*. Meropenem resistance was detected in 25.9, 85.5 and 37.0% of *P. aeruginosa*, *A. baumannii* and *K. pneumoniae* isolates, respectively. The AUC_0–24h,ss_ achieved with different polymyxin B dosing regimens (0.42–1.5 mg/kg/12 h) and infusion times (1 or 2 h) are shown in Supplementary Table S2. Steady-state exposures (range, 26.2–93.7 mg h/L) increased in proportion to the dose.

**TABLE 1:** Polymyxin B MIC distributions for *P. aeruginosa*, *A. baumannii* and *K. pneumoniae*.^a^

Strain (*n*)	MIC_50_ (mg/L)	MIC_90_ (mg/L)	MIC (mg/L) distribution (%)									
			0.125	0.25	0.5	1	2	4	8	16	32	>32
Pa (517)	1	1	0.39	0.58	7.74	87.8	1.93	1.35	0	0	0	0.19
Ab (262)	0.5	0.5	0	1.53	93.1	4.96	0	0	0	0	0	0.38
Kp (652)	0.5	0.5	0.46	26.1	65.5	4.91	1.84	0.15	0.61	0	0	0

a Resistance to meropenem (i.e., MIC ≥8 mg/L for *P. aeruginosa* and *A. baumannii*, MIC ≥4 mg/L for *K. pneumoniae*) was detected in 25.9% of *P. aeruginosa* isolates, 85.5% of *A. baumannii* isolates, and 37.0% of *K. pneumoniae* isolates.MIC_50_, the MIC value at which ≥50% of isolates are inhibited; MIC_90_, the MIC value at which ≥90% of isolates are inhibited.

### Pharmacokinetic/Pharmacodynamic Analysis of Polymyxin B With Different Dosing Regimens

**FIGURE 1:**
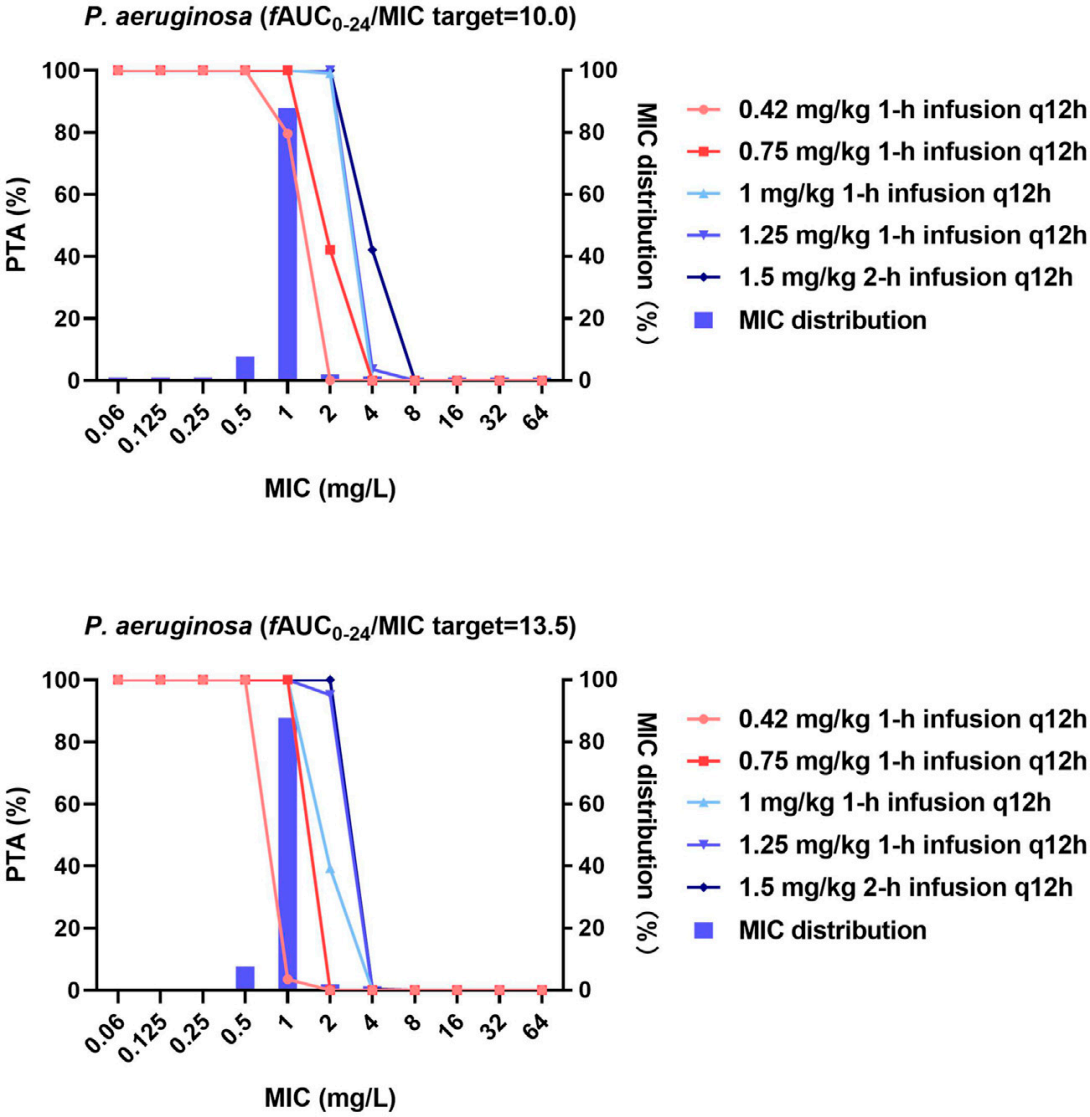
MIC distribution and PTA of different polymyxin B dosing regimens against *P. aeruginosa*. MICs of >32 mg/L are represented by 64 mg/L and PTA was determined using the AUC_0–24h_ at steady state. The *f*AUC_0–24h_/MIC targets of 10 and 13.5 represent 1-log_10_ CFU and 2-log_10_ CFU reductions, respectively.

**FIGURE 2:**
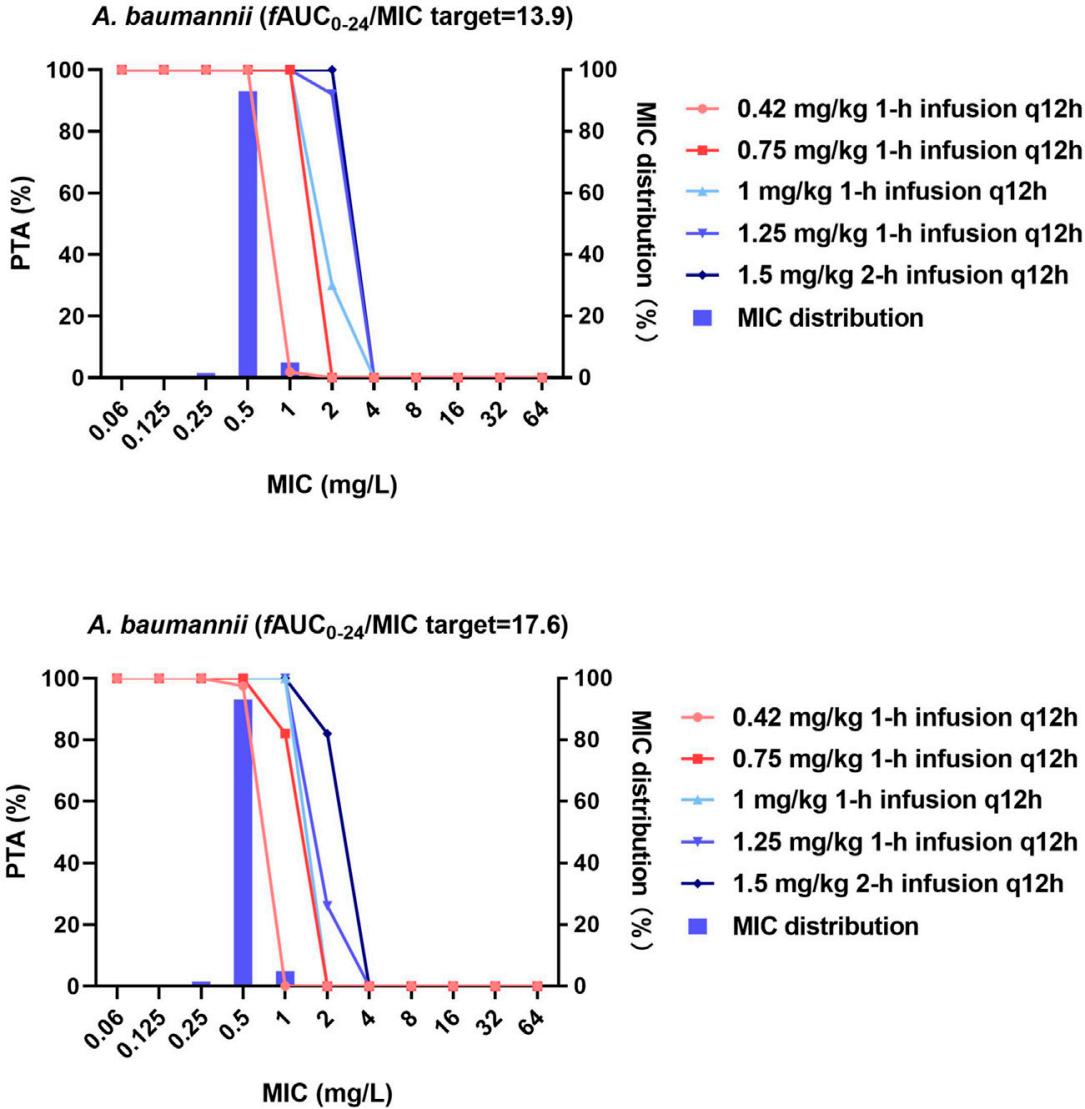
MIC distribution and PTA of different dosing regimens against *A. baumannii*. MICs of >32 mg/L are represented by 64 mg/L; PTA was determined using the AUC_0–24h_ at steady state. The *f*AUC_0–24h_/MIC targets of 13.9 and 17.6 represent 1-log_10_ CFU and 2-log_10_ CFU reductions, respectively.

**FIGURE 3:**
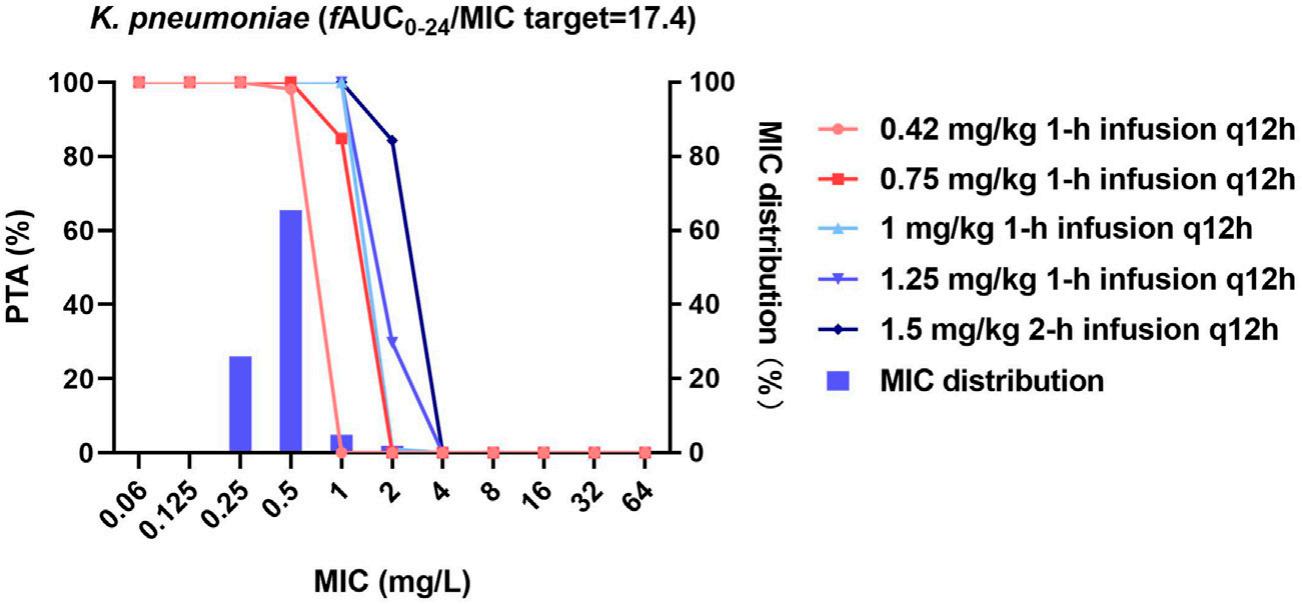
MIC distribution and PTA of different dosing regimens against *K. pneumoniae*. MICs of >32 mg/L are represented by 64 mg/L; PTA was determined using the AUC_0–24h_ at steady state. The *f*AUC_0–24h_/MIC target of 17.4 represents a 1-log_10_ CFU reduction.

Figures 1–3 and Supplementary Table S3 show the MIC distributions and PTA for each dosing regimen at steady state against strains of each bacterial species with diverse MICs. For *P. aeruginosa*, the *f*AUC_0–24h_/MIC required to achieve 1- and 2-log_10_ CFU killing (*f*AUC_0–24h_/MIC of 10 and 13.5, respectively) were achieved in 100% of cases for MICs ≤0.5 and ≤1 mg/L with the lower-dose regimens (0.42 and 0.75 mg/kg/12 h, respectively). However, with these regimens the PTA decreased dramatically as MICs increased above these values. For an MIC of 2 mg/L, the 1 mg/kg/12 h dosing regimen was effective with a PTA for 1-log_10_ CFU killing of 99% and the PTA for the 1.25 mg/kg/12 h dosing regimen was 95.1% to achieve 2- log_10_ CFU killing (Figure 1).

Against *A. baumannii*, both the 0.42 and 0.75 mg/kg/12 h regimens at steady state effectively achieved the *f*AUC_0–24h_/MIC target of 17.6 (2-log_10_ CFU reduction) against strains with MICs ≤0.5 mg/L (PTA = 97.5 and 100%), while the remaining dosing regimens were additionally effective against strains with an MIC ≤1 mg/L (PTA = 100%). No dosing regimen achieved the *f*AUC_0–24h_/MIC target of 17.6 when the MIC was ≥2 mg/L.

For *K. pneumoniae*, 0.42 and 0.75 mg/kg/12 h achieved the *f*AUC_0–24h_/MIC target of 17.4 in 98.1 and 100% of cases against strains with MICs ≤0.5 mg/L, respectively. All higher-dose regimens were additionally effective against strains with an MIC of ≤1 mg/L (PTA = 100%, Figure 3).

With the exception of the lowest dose regimen (0.42 mg/kg/12 h) against *P. aeruginosa*, all dosing regimens achieved a CFR >90% for all three bacterial species (MICs ranging from 0.125 to >32 mg/L; Table 2). The maximum treatable MICs for each bacterial species with the examined dosing regimens are shown in Table 3. Based on the dosing regimens of polymyxin B recommended throughout most of the world (1.5–2.5 mg/kg/day) and the highest *f*AUC_0–24h_/MIC target value, the PK/PD susceptibility breakpoints for *P. aeruginosa*, *A. baumannii* and *K. pneumoniae* were 2, 1 and 1 mg/L, respectively, for bloodstream infection. These breakpoints are higher than MIC_90_ values of current clinical isolates of all three bacterial species in China (Table 1), indicating the potential usefulness of polymyxin B for treatment of bloodstream infections caused by these bacteria.

**TABLE 2:** Cumulative fraction of response **(**CFR) to different dosing regimens of polymyxin B against *P. aeruginosa, A. baumannii* and *K. pneumoniae*.

Dose (mg/kg)	Infusion time (h)	Dosing frequency	CFR for different *f*AUC/MIC targets^a^ (%)				
			*P. aeruginosa* (517 isolates)		*A. baumannii* (262 isolates)		*K. pneumoniae* (652 isolates)
			Target = 10.0	Target = 13.5	Target = 13.9	Target = 17.6	Target = 17.4
0.42	1	q12h	78.8	12.3	94.8	92.3	90.9
0.75	1	q12h	97.3	96.5	99.6	98.5	96.7
1.0	1	q12h	98.2	97.1	99.6	99.6	97.4
1.25	1	q12h	98.8	98.6	99.5	99.5	97.8
1.5	2	q12h	98.9	98.2	99.5	99.5	99.1

a Targets are the median target values for 1-log_10_ CFU and 2-log_10_ CFU reductions in CFU killing in murine thigh infection models (Cheah et al., 2015; Landersdorfer et al., 2018).

**TABLE 3:** Susceptible breakpoints for isolates of *P. aeruginosa*, *A. baumannii* and *K. pneumoniae* based on the PK/PD of polymyxin B.

Dose (mg/kg)	Infusion time (h)	Dosing frequency	PK/PD breakpoint				
			*P. aeruginosa*		*A. baumannii*		*K. pneumoniae*
			MIC_50_ = MIC_90_ = 1 mg/L		MIC_50_ = MIC_90_ = 0.5 mg/L		
			Target = 10.0	Target = 13.5	Target = 13.9	Target = 17.6	Target = 17.4
0.42	1	q12h	0.5	0.5	0.5	0.5	0.5
0.75	1	q12h	1	1	1	0.5	0.5
1.0	1	q12h	2	1	1	1	1
1.25	1	q12h	2	2	2	1	1
1.5	2	q12h	2	2	2	1	1

## Discussion

Removal of the “susceptible” interpretation category for polymyxins by CLSI was driven primarily by lung infection data (He et al., 2013; Landersdorfer et al., 2018). However, polymyxins exhibit concentration-dependent killing against Gram-negative bacteria and efficacy is highly dependent upon concentrations achieved at the target site (Lee et al., 2019). For bloodstream infections, the average steady-state concentrations (*C*_ss,avg_) achieved with intravenously administered polymyxin B (0.45–3.38 mg/kg/day) or CMS (2–18 MIU/day) are 2–3 mg/L (polymyxin B and colistin) in critically-ill patients (Sandri et al., 2013; Nation et al., 2017). Thus, polymyxins may still be an appropriate therapeutic choice for bloodstream infections. The recent removal of the breakpoints for polymyxins by CLSI has caused significant uncertainties regarding their efficacy against bloodstream infections caused by the three aforementioned problematic Gram-negative bacteria. Therefore, we collected MIC data in the latest surveillance program in China and evaluated the rationality of the CLSI breakpoint modification for bloodstream infections.

In China, the MIC_90_ of polymyxin B for the three bacterial species were relatively low (1 mg/L for *P. aeruginosa* and 0.5 mg/L for *A. baumannii* and *K. pneumoniae*; Table 1) and the 0.75 mg/kg/12 h regimen was effective against >90% of pathogens. However, the success of this regimen will depend on regional susceptibility data. According to the SENTRY antimicrobial surveillance program for bloodstream infections (data collected from 45 nations including China between 1997 and 2016) (Diekema et al., 2019), the MIC_90_ of colistin for *P. aeruginosa* (7,107 isolates), *A. baumannii* or *A. calcoaceticus* (3,124 isolates) and *Enterobacteriaceae* (54,476 isolates) was 2, 2, and >4 mg/L, respectively. Given substantial regional susceptibility differences, effective therapeutic regimens must consider the regional susceptibility of the bacterial species involved.

In a recent Chinese study investigating the clinical efficacy and safety of polymyxin B-based regimens (100–200 mg/day, ≥5 days) for treatment of bloodstream infection caused by extensively-drug resistant Gram-negative bacteria, the clearance rate of microorganisms was 65.2%, overall effectiveness (cure or improvement) 59.0%, and 28-day all-cause mortality 41.0% (Zhao et al., 2020). Importantly, both effectiveness and microbial clearance were significantly higher with higher daily doses of polymyxin B (150 and 200 mg) compared to the lowest daily dose (100 mg). This is in agreement with other clinical data which support the use of high doses of polymyxin B for bloodstream infections (Elias et al., 2010; Cai et al., 2020).

The recommended dose of intravenously administered polymyxin B throughout much of the world is 1.5–2.5 mg/kg/day with a loading dose of 2–2.5 mg/kg (Tsuji et al., 2019). In China, the only brand of polymyxin B currently available has a recommended intravenous dose of 50–100 mg/day (Polymyxin B for injection (Daily Med, 2017), equivalent to 0.83–1.66 mg/kg/day (0.42–0.83 mg/kg/12 h) for a 60 kg patient. Importantly, Lakota et al. (2018) proposed that for pathogens with MICs ≤2 mg/L, the target AUC_0–24h,ss_ for polymyxin B should be 50–100 mg h/L. In our study, the polymyxin B AUC_0–24h,ss_ achieved with the 0.42 and 0.75 mg/kg/12 h dosing regimens fell below this target range (AUC_0–24h,ss_ of 26.2 and 46.9 mg h/L, respectively; Supplementary Table S2), indicating that the recommended dose in China is very likely insufficient. Loading doses have no impact on the exposure at steady state but in favour of the rapid achievement to the effective concentrations at day 1. From our previous population pharmacokinetic analysis, gender and age are covariates in the final model (Liu et al., 2021). However, both of them had no significant effect on the AUC_0–24h,ss_. If the new CLSI breakpoints (M100-S30, 2020) are employed, the recent MIC data collected in Chinese hospitals showed that most isolates of *P. aeruginosa*, *A. baumannii*, and *K. pneumoniae* (MIC_90_ values of 1, 0.5, and 0.5 mg/L, respectively) would have intermediate susceptibility to polymyxin B. However, further PK/PD analysis showed that increasing doses could be effective against strains with MICs up to 2 mg/L. Taking decreases in bacterial counts of 1-log_10_ CFU (for *K. pneumoniae*) or 2-log_10_ CFU (for *P. aeruginosa* and *A. baumannii*) kill as the target values, dosing regimens of ≥0.75 mg/kg/12 h could be effective against *P. aeruginosa* with MIC of 1 mg/L; for *A. baumannii* and *K. pneumoniae*, all dosing regimens were effective against isolates with MIC ≤0.5 mg/L. One PK/PD study on polymyxin B for bloodstream infections has been conducted though the rationality of the present CLSI breakpoints were not well explained which is a valuable reference in clinical practice (Wu et al., 2021). In addition, the possible effects from variabilities of protein binding and PK/PD targets were not fully investigated. Based on the recommended polymyxin B dose of 1.5–2.5 mg/kg/day, we have proposed here PK/PD susceptibility breakpoints for *P. aeruginosa*, *A. baumannii* and *K. pneumoniae* of 2, 1 and 1 mg/L, respectively. Thus, EUCAST interpretation standards would be recommended in terms of PK/PD. Given strain-to-strain variability in PD targets, we conducted sensitivity analysis using different PK/PD targets (7.4 for *P. aeruginosa* and *A. baumannii* and 28.0 for *K. pneumoniae*). The breakpoints for *P. aeruginosa*, *A. baumannii* and *K. pneumoniae* were 2, 2 and 1 mg/L, respectively. The variable protein binding may affect the PK/PD breakpoints as well. According to the reported protein binding of 78.5–92.4% (Zavascki et al., 2008), the breakpoints were 1, 0.5, 0.5 mg/L assuming protein binding rate was 78.5% and 0.25, 0.25, 0.25 mg/L using the value of 92.4% for *P. aeruginosa*, *A. baumannii* and *K. pneumoniae*. The protein binding of less than 80% is acceptable for treating infections caused by these three bacterial species. Clearly, for all three pathogens polymyxin B would not be recommended for the treatment of bloodstream infections when the pathogen MIC is >2 mg/L.

Xie et al. compared the efficacy and safety of polymyxin B in various patient populations (Xie et al., 2020). Exposure was noticeably influenced by patient’ body weight when doses were calculated in mg/kg. The lower exposures achieved in patients with less body weight such as 50 kg in comparison to patients with body weight of 75–100 kg are more likely to result in treatment failure. This is important to note the recommended dose of polymyxin B in China (50–100 mg/day) is not based on body weight. The lower end of the recommended dosing range in most Western countries (1.5 mg/kg/day) equals to 90 mg/day for a 60 kg patient. Utilizing polymyxin B over 90 mg/kg is essential in terms of PK/PD.

In summary, the determination of breakpoints should be species- and infection-site specific and underpinned by solid PK/PD data. Given the polymyxins remain important last-line antibiotics for the treatment of infections caused by carbapenem-resistant Gram-negative bacteria, appropriate PK/PD considerations must be given when determining their breakpoints and dosage regimens. Adequate doses (i.e., 90–100 mg/day) should be utilized in terms of Chinese recommended dosing range. The revival of CLSI susceptibility breakpoint of polymyxins for bloodstream infections is potential in the future.

## Data Availability

The original contributions presented in the study are included in the article/Supplementary Material, further inquiries can be directed to the corresponding authors.
