# Supplementary material for: Pharmacokinetic/Pharmacodynamic Based Breakpoints of Polymyxin B for Bloodstream Infections Caused by Multidrug-Resistant Gram-Negative Pathogens
Source: Front Pharmacol. 2022 Jan 4;12:785893. doi: 10.3389/fphar.2021.785893 (PMC8763792; doi:10.3389/fphar.2021.785893)
Supplement: Supplementary file 1 [file DataSheet1.zip › Table 1.DOCX]

**Table S1.** Polymyxin B PK/PD targets for 1- and 2-log_10_ CFU killing of *P. aeruginosa*, *A. baumannii* and *K. pneumoniae*.

| **Strain^#^** | **Animal model** | **PK/PD index** | **PK/PD target^†^** | | **Reference** |
| --- | --- | --- | --- | --- | --- |
|  |  |  | **1-log_10_ CFU killing (median)** | **2-log_10_ CFU killing (median)** |  |
| *P. aeruginosa** | Murine thigh infection model | *f*AUC_0‑24h_/MIC | 8.8-11.5 (10) | 11.5-16.4 (13.5) | Cheah, S.E., 2015 |
| *A. baumannii** |  |  | 13.1-14.6 (13.9) | 16.7-18.4 (17.6) | Cheah, S.E., 2015 |
| *K. pneumoniae* |  |  | 3.72-28.0 (17.4) | - | Landersdorfer, C.B., 2018 |

^†^The PK/PD target utilized was the area under the unbound concentration-time curve over 24 h to the MIC ratio (*f*AUC/MIC). This index has been shown to most closely correlate with polymyxin bacterial killing (Bergen, P.J., 2008, Bergen, P.J., 2010, Cheah, S.E., 2015, Landersdorfer, C.B., 2018, Tam, V.H., 2005).

^#^ For PTA analysis, PK/PD index target values from *P. aeruginosa* 19056 and *A. baumannii* N-16870.213 (median and IQR) were utilized. For, *K. pneumoniae*, as PK/PD target values for three different strains were reported without confidence intervals, the median values of the PK/PD target values of each strain were used to attain the median and range.

* PK/PD targets determined using colistin (polymyxin E). Polymyxin B and colistin have essentially identical *in vitro* potencies (as measured by MICs) and spectra of activity against common Gram-negative organisms (Gales, A.C., CHINET, 2020). Landersdorfer *et al*. demonstrated no difference in the magnitude of bacterial killing between polymyxin B and colistin in the thigh infection model.

- Dosing regimens were unable to produce a 2-log_10_ CFU decrease.

The median target values for 1-log_10_ and 2-log_10_ reductions in CFU killing were used for PK/PD analysis, i.e., against *P. aeruginosa*, *f*AUC_0-24h_/MICs of 10 and 13.5 were required for a 1-log_10_ CFU killing and 2-log_10_ CFU killing decrease, respectively.

**Table S2.** AUC_0-24h,ss_ achieved with different dosing regimens of polymyxin B.

| **No** | **Dose (mg/kg)** | **Infusion time (h)** | **Dosing frequency** | **Treatment duration (d)** | **AUC_0-24h,ss_**  **(mg·h/L)** | **SD** |
| --- | --- | --- | --- | --- | --- | --- |
| 1* | 0.42 | 1 | q12h | 7 | 26.2 | 3.0 |
| 2 | 0.75 | 1 | q12h | 7 | 46.9 | 5.4 |
| 3 | 1.0 | 1 | q12h | 7 | 62.8 | 7.2 |
| 4 | 1.25 | 1 | q12h | 7 | 78.3 | 8.9 |
| 5 | 1.5 | 2 | q12h | 7 | 93.7 | 10.6 |

* The dosing regimen of 0.42 mg/kg/12h is derived from the 50 mg/day dose for a ~60-kg patient recommended in the polymyxin B package insert (Shanghai First Biochemical Pharmaceutical Co., Ltd.). SD, standard deviation.

AUC_0-24h,ss_, the area under the concentration-time curve across 24 h at steady state.

**References**

Bergen, P.J., Bulitta, J.B., Forrest, A., Tsuji, B.T., Li, J., and Nation, R.L. (2010). Pharmacokinetic/pharmacodynamic investigation of colistin against *Pseudomonas aeruginosa* using an *in vitro* model. *Antimicrob Agents Chemother* 54(9)**,** 3783-3789. doi: 10.1128/AAC.00903-09.

Bergen, P.J., Li, J., Nation, R.L., Turnidge, J.D., Coulthard, K., and Milne, R.W. (2008). Comparison of once-, twice- and thrice-daily dosing of colistin on antibacterial effect and emergence of resistance: studies with *Pseudomonas aeruginosa* in an *in vitro* pharmacodynamic model. *J Antimicrob Chemother* 61(3)**,** 636-642. doi: 10.1093/jac/dkm511.

Cheah, S.E., Wang, J., Nguyen, V.T., Turnidge, J.D., Jian, L., and Nation, R.L. (2015). New pharmacokinetic/pharmacodynamic studies of systemically administered colistin against *Pseudomonas aeruginosa* and *Acinetobacter baumannii* in mouse thigh and lung infection models: smaller response in lung infection. *J Antimicrob Chemother* 70(12)**,** 3291-3297. doi: 10.1093/jac/dkv267.

CHINET. (2020). http://www.chinets.com/Data/AntibioticDrugFast [Accessed October 15, 2020]

Gales, A.C., Jones, R.N., and Sader, H.S. (2011). Contemporary activity of colistin and polymyxin B against a worldwide collection of Gram-negative pathogens: results from the SENTRY Antimicrobial Surveillance Program (2006-09). *J Antimicrob Chemother* 66(9)**,** 2070-2074. doi: 10.1093/jac/dkr239.

Landersdorfer, C.B., Wang, J., Wirth, V., Chen, K., Kaye, K.S., Tsuji, B.T., et al. (2018). Pharmacokinetics/pharmacodynamics of systemically administered polymyxin B against *Klebsiella pneumoniae* in mouse thigh and lung infection models. *J Antimicrob Chemother* 73(2)**,** 462-468. doi: 10.1093/jac/dkx409.

Tam, V.H., Schilling, A.N., Vo, G., Kabbara, S., Kwa, A.L., Wiederhold, N.P., et al. (2005). Pharmacodynamics of polymyxin B against *Pseudomonas aeruginosa*. *Antimicrob Agents Chemother* 49(9)**,** 3624-3630. doi: 10.1128/AAC.49.9.3624-3630.2005.
